# A R2R3 MYB Transcription Factor, *TaMYB391*, Is Positively Involved in Wheat Resistance to *Puccinia striiformis* f. sp. *tritici*

**DOI:** 10.3390/ijms232214070

**Published:** 2022-11-15

**Authors:** Mehari Desta Hawku, Fuxin He, Xingxuan Bai, Md Ashraful Islam, Xueling Huang, Zhensheng Kang, Jun Guo

**Affiliations:** 1 State Key Laboratory of Crop Stress Biology for Arid Areas, College of Plant Protection, Northwest A&F University, Xianyang 712100, China; 2 Department of Crop Science, College of Agriculture, Animal Science and Veterinary Medicine, University of Rwanda, Musanze P.O. Box 210, Rwanda

**Keywords:** MYB, *Puccinia striiformis* f. sp. *tritici*, *Triticum aestivum*, resistance, hypersensitive response

## Abstract

A biotrophic fungus, *Puccinia striiformis* f.sp. *tritici (Pst)*, which causes stripe rust disease in wheat is the most yield-limiting factor in wheat production. Plants have complex defense mechanisms against invading pathogens. Hypersensitive response (HR), a kind of programmed cell death (PCD) at the infection site, is among these defense mechanisms. Transcription factors (TFs) play a crucial role in plant defense response against invading pathogens. Myeloblastosis (MYB) TFs are among the largest TFs families that are involved in response to both biotic and abiotic stresses. However, little is known about the mechanisms of MYB TFs during the interaction between wheat and the stripe rust fungus. Here, we identified an R2R3 MYB TF from wheat, designated as *TaMYB391,* and characterized its functional role during wheat–*Pst* interaction. Our data indicated that *TaMYB391* is induced by *Pst* infection and exogenous application of salicylic acid (SA) and abscisic acid (ABA). *TaMYB391* is localized in the nucleus of both wheat and *Nicotiana benthamiana*. Transient overexpression of *TaMYB391* in *N. benthamiana* triggered HR-related PCD accompanied by increased electrolyte leakage, high accumulation of reactive oxygen species (ROS), and transcriptional accumulation of SA defense-related genes and HR-specific marker genes. Overexpression of *TaMYB391* in wheat significantly enhanced wheat resistance to stripe rust fungus through the induction of pathogenesis-related (PR) genes, ROS accumulation and hypersensitive cell death. On the other hand, RNAi-mediated silencing of *TaMYB391* decreased the resistance of wheat to *Pst* accompanied by enhanced growth of the pathogen. Together our findings demonstrate that *TaMYB391* acts as a positive regulator of HR-associated cell death and positively contributes to the resistance of wheat to the stripe rust fungus by regulating certain PR genes, possibly through SA signaling pathways.

## 1. Introduction

Wheat (*Triticum aestivum*) is one of the most important stable crops globally. Its production and productivity, however, are influenced by both biotic and abiotic stresses. Stripe rust disease which is caused by a biotrophic fungus called *Puccinia striiformis* f. sp. *tritici* (*Pst*) is the most yield-limiting factor in wheat production. The disease occurs in more than 60 countries worldwide, and stripe rust epidemics occur more frequently and cause annual crop losses of 5–10% in several countries [1].

Plants have complex defense mechanisms against invading pathogens. Upon invasion by pathogens, pattern-triggered immunity (PTI) and effector-triggered immunity (ETI) are triggered by the recognition of pathogen-associated molecular patterns (PAMPs) and effectors, respectively [2]. Both PTI and ETI lead to the activation of multiple defense signal transduction pathways, involving salicylic acid (SA), reactive oxygen species (ROS), and mitogen-activated protein kinase (MAPK) [3]. These defense responses involve the rapid transcriptional reprogramming mediated by transcription factors (TFs), such as the upregulation of defense genes encoding antimicrobial proteins [4]. These eventually result in plant resistance against plant pathogens [2,4]. Several TFs have been reported to play a crucial role in wheat defense responses to *Pst*. For instance, *TaBZR2*, *TaLOL2*, *TabZIP74*, *TaAP2-15*, *TaWRKY10* and *TaCBF1* act as positive regulators in wheat stripe rust resistance [5,6,7,8,9,10]. In addition, previous studies have reported that wheat TFs such as *TaWRKY62* and *TaWRKY70* were positively involved in high-temperature seedling plant resistance to *Pst* [11,12].

Myeloblastosis (MYB) TFs are among the largest TF families. The conserved MYB DNA-binding domain comprises 1–4 tandem incomplete repeats of MYB repeats, and each incomplete repeat contains 50–53 amino acid residues [13]. Based on the number of adjacent imperfect repeats of these domains, MYB TFs are classified into four categories: 1R-MYB/MYB-related, R2R3-MYB, 3R-MYB and 4R-MYB [14]. In recent years, MYB TF genes have been identified and characterized in the genomes of many plant species, including *Arabidopsis*, *Gossypium hirsutum*, *Dendrobium officinale*, *Oryza sativa*, *Arachis hypogaea*, *Rhododendron delavayi* and *Triticum aestivum* [13,15]. There are some differences in the number of different types of MYB in plants, and most of the identified MYB proteins belong to the R2R3-MYB subgroup [13]. Many MYB proteins have been reported to play significant roles in diverse biological processes and defense responses to environmental stresses [13,14,16]. Some of the reported MYB proteins are involved in defense response against invading pathogens. For instance, overexpression of *AtMYB96*, an R2R3-MYB gene, increased disease resistance to pathogen infection in *Arabidopsis* [17]. In *Arabidopsis*, disease resistance in *myb15* mutant plants was reduced after inoculation with the avirulent bacterial pathogen, *Pseudomonas syringae* pv. *tomato* DC3000 (*AvrRpm1*) [18]. Overexpression of *MYB115* from poplar showed increased resistance to the fungal pathogen, *Dothiorella gregaria* [19]. In rose, the silencing of *RcMYB84* and *RcMYB123* increased the susceptibility of plants to *Botrytis cinerea* [20]. Ectopic expression of the *Thinopyrum intermedium TiMYB2R-1* in wheat significantly improved wheat resistance to take-all disease, caused by *Gaeumannomyces graminis* [21]. In Einkorn wheat, decreased expression of *TuMYB46L*, a R2R3-MYB gene, induced an elevated function of the 1-aminocyclopropane-1-carboxylic acid oxidase gene *TuACO3* and promoted wheat defense against *Blumeria graminis* f.sp. *tritici* (*Bgt*) [22]. In wheat, overexpression of two MYB genes, *TaPIMP1* [23] and *TaPIMP2* [24] significantly increased resistance to the fungal pathogen *Bipolaris sorokiniana*. Overexpression of *TaRIM1*, a R2R3-MYB gene from wheat, positively modulated wheat defense response to *Rhizoctonia cerealis* [25]. The silencing of three MYB genes, *TaMYB4*, *TaMYB29* and *TaLHY* from wheat increased susceptibility to *Pst* [26,27,28]. Although several MYB transcription factors involved in defense response have been identified in wheat, the functions of MYB genes during the interaction between wheat and *Pst* need to be studied in depth.

A number of phytohormones have been reported to play a significant role in mediating defense responses. Any change in the status of these hormones, their perception and signaling results in the alteration of defense responses to pathogens [29,30]. Upon invasion by pathogens, plants accumulate high amounts of SA that are involved in the activation of defense responses, such as the induced expression of pathogenesis-related (PR) genes [31]. SA plays a significant role in the plant–pathogen interactions. Mutants of an isochorismate synthase gene, *ICS1*, which is a key enzyme for pathogen-induced SA biosynthesis, showed enhanced susceptibility to pathogen infection and were unable to accumulate high SA levels, failing to develop systemic acquired resistance (SAR) [32]. In plants, most SA exists in a glucose-conjugated inactive form. Thus, the defense response was enhanced by inhibiting the conversion of SA to SA-O-β-D-glucoside [30,33]. Mutants of salicylic acid 5-hydroxylase gene, *S5H*, which encodes a key enzyme that catalyzes the conversion of SA to 2,5-dihydroxybenzoic acid (2,5-DHBA), exhibit higher SA content and a constitutive defense response [34]. In plants, SA-related signaling is required for PTI and ETI, and is involved in controlling cell death during ETI [32].

ETI is usually correlated with ROS accumulation, which ultimately leads to the activation of various groups of defense-related genes [2]. ROS as the signaling molecules of the secondary signaling process are well known for their roles in response to environmental stresses [35]. ROS molecules have been shown to play an important role in triggering the HR [36]. HR, where necrotic lesions develop at the spot where the pathogen enters the host, is the most visible phenomenon of the ETI [37]. Precise regulation of organelle-specific ROS is essential for a successful immune response [38].

In this study, we reported the functional role of a wheat R2R3 MYB TF, *TaMYB391*, during the interaction between wheat and the stripe rust fungus. We found that *TaMYB391* is highly induced upon *Pst* inoculation and exogenous hormone treatment. The sub-cellular localization assays revealed that *TaMYB391* is localized to the nucleus of both wheat and *Nicotiana benthamiana*. *Agrobacterium*-mediated transient overexpression of *TaMYB391* in *N. benthamiana* induced programmed cell death accompanied by increased ROS accumulation and transcriptional induction of HR-specific and defense-related marker genes. Results obtained from both overexpression and silencing (RNAi) experiments demonstrated that *TaMYB391* is positively involved in the defense responses of wheat against *Pst* infection. Our findings provide new insights into understanding the molecular mechanisms of MYB TFs.

## 2. Results

### 2.1. Identification and Sequence Analysis of TaMYB391

The amino acid sequences of an *Arabidopsis* gene, *AtMYB44*, which is involved in resistance response to biotrophic pathogen and regulates the expression of *WRKY70* [39] were used as a query search, and we found a homologous protein (TraesCS7D02G514800) in wheat, which was previously named as TaMYB391 [38]. *TaMYB391* was then isolated from wheat cv. Su11 infected with *Pst* isolates. The coding sequence (CDS) is 1077 bp in length. The predicted ORF of *TaMYB391* encodes 358 deduced amino acids with a molecular weight of 38.85 kDa and an isoelectric point (pI) of 8.07. Multisequence alignment indicated that *TaMYB391* is highly conserved with *Oryza sativa* (GenBank accession No. XP_015641187.1), *Brachypodium distachyon* (GenBank accession No. XP_024318409.1), *Hordeum vulgare* (GenBank accession No. KAE8776319.1) and *Sorghum bicolor* (GenBank accession No. XP_002451692.1) (Figure S1a). Domain feature analysis revealed that *TaMYB391* contains two MYB DNA binding domains: R2 (amino acid 73-118) and R3 (amino acid 125-167) (Figure S1a). Eight MYB amino acid sequences from different plant species were downloaded from GenBank, and a phylogenetic tree was constructed using the neighbor-joining analysis method. Phylogenetic analysis indicated the homology similarity between TaMYB391 and MYB proteins from other plant species. As indicated in Figure S1b, TaMYB391 is closely related to OsMYB77 and HvMYB44.

### 2.2. Transcriptional Responses of TaMYB391 to Pst

**Figure 1:**
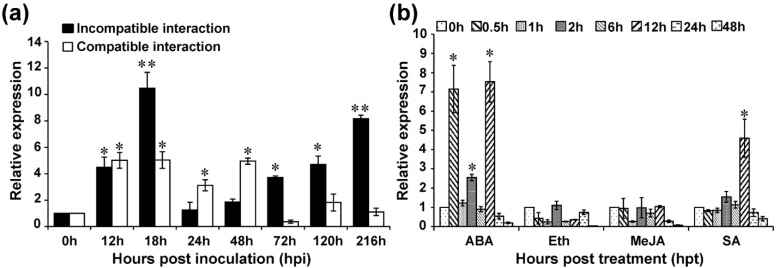
*TaMYB391* is induced upon *Pst* infection and exogenous application of hormones. (**a**) *TaMYB391* is induced in wheat leaves upon infection with *Pst* isolates, CYR23 (incompatible) and CYR31 (compatible) during a 216 h-long time course. (**b**) Exogenous application of hormones in wheat leaves induced the expression of *TaMYB391*. Two-week-old wheat seedlings were used in this experiment. Wheat leaves treated with distilled water or 0.1% ethanol were included as a control. *TaEF-1α* was used as an internal reference. The relative quantity of expression of *TaMYB391* was computed via the comparative threshold (2^−ΔΔCt^) method. The transcript levels were quantified by RT-qPCR and the values were standardized to that of *TaEF-1α* and presented as relative changes to the control. The expression level of *TaMYB391* at time 0 h was normalized as 1. Error bars represent the variation among three independent replicates. Statistical variations were analyzed using Student’s *t*-test. *, *p* < 0.05, **, *p* < 0.01.

To gain insight into whether *TaMYB391* participates in the wheat response to *Pst* infection, the transcript levels of *TaMYB391* in infected leaves at different time points were analyzed using RT-qPCR. As the cultivar Suwon11 was reported to have a resistant gene, *YrSu*, and be resistant to CYR23 (incompatible) and highly susceptible to CYR31 (compatible) [40], the transcript levels of *TaMYB391* were analyzed in both compatible and incompatible interactions. In the incompatible interaction, transcript levels were upregulated at 12, 18, 72, 120 and 216 h post-inoculation (hpi) as compared to the control. Upon infection with the avirulent race CYR23, the transcript levels were induced as early as 12 hpi. The highest transcription accumulation was obtained at 18 hpi which was ~10-fold higher than the control. The next highest transcript accumulation was attained at 216 hpi which was ~8-fold higher compared to the control (Figure 1a). The transcript profiles of *TaMYB391* in the compatible interaction were also significantly upregulated at 12, 18, 24 and 48 hpi as compared to the control (Figure 1a). These results suggested that *TaMYB391* may be involved in wheat resistance response against *Pst* during their interaction.

### 2.3. Transcriptional Response of TaMYB391 to Exogeneous Hormones

The expression patterns of *TaMYB391* in response to four hormones (ABA, Eth, MeJA and SA) were investigated using RT-qPCR. As illustrated in Figure 1b, the expression levels of *TaMYB391* after treatment with ABA were induced ~7-fold as early as 0.5 h post-treatment (hpt). As compared to the controls, maximum transcript levels of *TaMYB391* (~7.5-fold) were obtained at 12 hpt. After treatment with SA, the transcript levels of *TaMYB391* were significantly upregulated at 12 hpt which was ~4.5-fold compared to control plants. On the other hand, when treated with MeJA and Eth, the expression of *TaMYB391* was repressed at most of the time points analyzed (Figure 1b). These results suggest that *TaMYB391* might participate in diverse hormone signal transduction pathways.

### 2.4. TaMYB391 Is Localized in the Nucleus

We verified the sub-cellular localization of TaMYB391 in both wheat mesophyll protoplasts and *N. benthamiana* cells. We transiently expressed the constructs: p16318hGFP:*TaMYB391* and pCAMBIA1302:*TaMYB391*-GFP (green fluorescence protein) or empty vector p16318hGFP as a control in wheat mesophyll protoplasts and *N. benthamina* through PEG- and *Agrobacterium tumefaciens*-mediated transformation, respectively. As illustrated in Supplementary Figure S2a, TaMYB391-GFP fusion protein was localized in the nucleus of wheat mesophyll cells, while the GFP protein produced by protoplasts transformed with the empty vector was detected throughout the cell. Similar results were obtained in *N. benthamiana* (Figure S2b). These results indicated that TaMYB391 is a nuclear-localized protein.

### 2.5. Transient Overexpression of TaMYB391 in N. benthamiana Triggered HR-Related Programmed Cell Death

**Figure 2:**
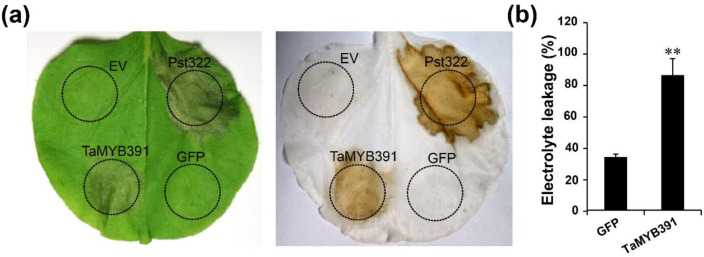
Transient overexpression of *TaMYB391* induces programmed cell death in *N. benthamiana*. (**a**) *A. tumefaciens* cells carrying *TaMYB391*, *Pst332*, or an empty vector were infiltrated into *N. benthamiana* leaves within the regions indicated by dashed lines. The cell death phenotype was photographed seven days after agroinfiltration. The right panel shows the same leaf as on the left panel after decolorization with ethanol. (**b**) Quantification of cell death by measuring electrolyte leakage four days post-agroinfiltration (dpa). Means and SEs were computed from three independent experiments. The statistical analyses were performed with Student’s *t*-test. Bars indicate ± SE. **, *p* < 0.01.

In plant immunity, PCD plays a significant role against invading pathogens and abiotic stress [37]. To determine whether *TaMYB391* is involved in producing PCD, we transiently overexpressed *TaMYB391* in *N. benthamiana* using a PVX vector. Four- to six-week-old tobacco leaves were agroinfiltrated with the following recombinants: PVX: *TaMYB391*, PVX: *Pst322* (positive control), GFP and empty vector (EV) as negative controls. After seven days, obvious cell death was observed on PVX: *TaMYB391*- and PVX: *Pst322*-infiltrated leaves (Figure 2a), suggesting their role in cell death induction, whereas there was no cell death on GFP- or empty vector-infiltrated leaves. The cell death was further confirmed by electrolyte leakage. A high percentage of electrolyte leakage was obtained from PVX: *TaMYB391*-infiltrated leaves compared to control leaves (Figure 2b). These results strongly suggested that *TaMYB391* might be involved in producing PCD.

### 2.6. TaMYB391 Activates Plant Immunity Responses

In plant defense response, programmed cell death, usually known as HR, is produced when plants recognize effectors and develop ETI which is usually associated with ROS accumulation and induction of defense-related genes [2,36]. To examine whether the *TaMYB391*-triggered cell death is associated with plant innate immunity response, ROS accumulation and transcriptional accumulation of HR-specific marker genes, *NbHSR203J* and *NbHIN1* [41,42], were determined in *N. benthamiana*. At 24 h after infiltration, there was no ROS detected in the control plants, whereas obvious ROS accumulation was observed in *TaMYB391*-infiltrated leaves (Figure S3a). Furthermore, the expression levels of *NbHSR203J* and *NbHIN1* were significantly activated by *TaMYB391* at different time-courses (Figure S3b). These results suggested that *TaMYB391* is involved in the activation of plant immunity responses.

To further verify whether *TaMYB391*-triggered immunity is related with salicylic acid (SA) signaling pathways, we analyzed the expression levels of defense-related genes, *NbPR1a* and *NbPR2* [43] which are well known for their SA-dependent expression. The transcriptional accumulation of these genes was analyzed through RT-qPCR. Their transcriptional abundance in the *TaMYB391* agroinfiltrated plants was indicative of a significant upregulation through different time points compared with the controls (Figure S3b). These results suggested that *TaMYB391* could activate innate immunity through the activation of SA-mediated defense pathways.

### 2.7. Overexpression of TaMYB391 in Wheat Enhances Wheat Resistance to Pst Infection

**Figure 3:**
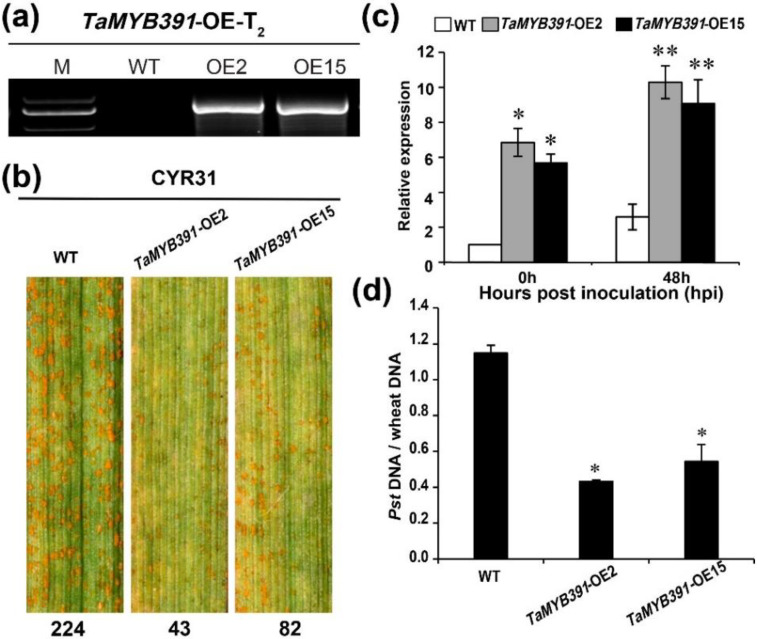
Overexpression of *TaMYB391* in wheat enhances wheat resistance to *Puccinia striiformis* f.sp. *tritici* infection. (**a**) Two overexpression lines, *TaMYB391*-OE2 and *TaMYB391*-OE15 validated via PCR in the T_2_ generation. M: molecular marker; WT: wild type. (**b**) Phenotype of *TaMYB391*-OE2 and *TaMYB391*-OE15 inoculated with the virulent *Pst* race CYR31. Numbers below the leaves represent the number of uredia quantified using ImageJ software. (**c**) Expression patterns of *TaMYB391* in the *TaMYB391*-OE2 and *TaMYB391*-OE15 lines when inoculated with CYR31. (**d**) Fungal and wheat biomass ratio quantified from total genomic DNA content at 14 dpi. WT was used as a control. The transcript level of genes in control plants at 0 hpi was standardized as 1. Values represent the means ± SEs (*n* = 3). Significant variations between transformed and control plants at the same time points were computed by Student’s *t*-test and indicated by asterisks. *, *p* < 0.05; **, *p* < 0.01.

To validate the functional role of *TaMYB391* during the wheat–*Pst* interaction, *TaMYB391*-overexpression transgenic wheat plants were developed by using wheat cultivar Fielder as receptor material. Five successfully transformed T_1_ transgenic lines (OE2, OE4, OE11, OE12 and OE15) were detected via PCR using specific primers (Figure S4a,b and Table S1). Two transgenic lines (OE2 and OE15) were selected for further study based on their higher transcript levels analyzed through RT-qPCR (Figure S4c). The T_2_ transgenic lines (OE2 and OE15) were further validated by PCR (Figure 3a), and their defense response was evaluated following inoculation with the virulent *Pst* race CYR31. At 14 days post-inoculation (dpi), more uredia were observed on the wild-type (WT) Fielder plants (224 per leaf area) compared to the transgenic lines (43 and 82 per leaf area, respectively) (Figure 3b). RT-qPCR analysis showed that the transcript levels of *TaMYB391* in the transgenic lines were significantly higher than those recorded in WT controls (Figure 3c). Furthermore, biomass analysis showed that significantly lower fungal biomass was obtained in the overexpressed transgenic lines compared to the untransformed WT (Figure 3d). These results revealed that the overexpression of *TaMYB391* enhanced wheat resistance to *Pst* infection.

### 2.8. RNAi-Mediated Silencing of TaMYB391 Impairs Wheat Resistance to Pst Infection

**Figure 4:**
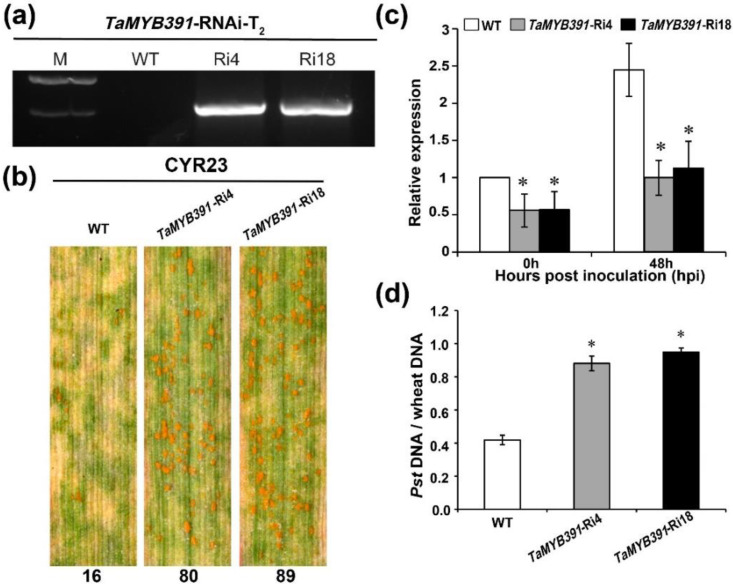
RNAi-mediated silencing of *TaMYB391* enhances wheat susceptibility to *Puccinia striiformis* f.sp. *tritici* infection. (**a**) RNAi lines in the T_2_ generation, *TaMYB391*-Ri4 and *TaMYB391*-Ri18 validated via PCR. M, molecular marker; WT, wild type. (**b**) Phenotype of *TaMYB391*-Ri4, *TaMYB391*-Ri18 and WT plants inoculated with the avirulent *Pst* race CYR23. Numbers below the leaves represent the number of uredia quantified using ImageJ software. (**c**) Expression patterns of *TaMYB391* in *TaMYB391*-Ri4 and *TaMYB391*-Ri18 lines when inoculated with CYR23. (**d**) Fungal and wheat biomass ratio quantified from total genomic DNA content at 14dpi. WT plants were used as controls. The transcript level of genes in control plants at time 0 hpi was standardized as 1. Values represent the means ± SEs (*n* = 3). Significant variations between transformed and control plants at the same time points were computed by Student’s *t*-test and indicated by asterisks. *, *p* < 0.05.

To further verify the functional role of *TaMYB391* in wheat defense against *Pst* by loss-of-function approach, we generated transgenic wheat plants under-expressing the *TaMYB391* gene via RNAi. Five successfully transformed *TaMYB391*-RNAi transgenic lines (Ri3, Ri4, Ri9, Ri18, Ri26) in the T_1_ generation were validated by PCR using universal primers (Figure S5a,b and Table S1), and those lines showing the lowest transcriptional accumulation (Ri4 and Ri18) were selected for further study (Figure S5c). In the T_2_ generation, these lines were again verified by PCR (Figure 4a), and their defense response was evaluated following inoculation with the avirulent *Pst* race CYR23. At 14 dpi, obvious HR symptoms were observed on all leaves. The two *TaMYB391*-RNAi lines had more uredia (80 and 89 per leaf area, respectively) than the WT plants (16 per leaf area) (Figure 4b). RT-qPCR analysis indicated that the transcript abundance of *TaMYB391* in the RNAi lines was significantly decreased compared to WT (Figure 4c). Furthermore, fungal biomass was significantly increased in *TaMYB391*-RNAi plants compared to WT (Figure 4d). These results suggested that *TaMYB391* positively contributes to wheat resistance to *Pst*.

### 2.9. TaMYB391 Positively Regulates the Expression of Pathogenesis-Related (PR) and ROS-Scavenging Genes during Pst Infection

**Figure 5:**
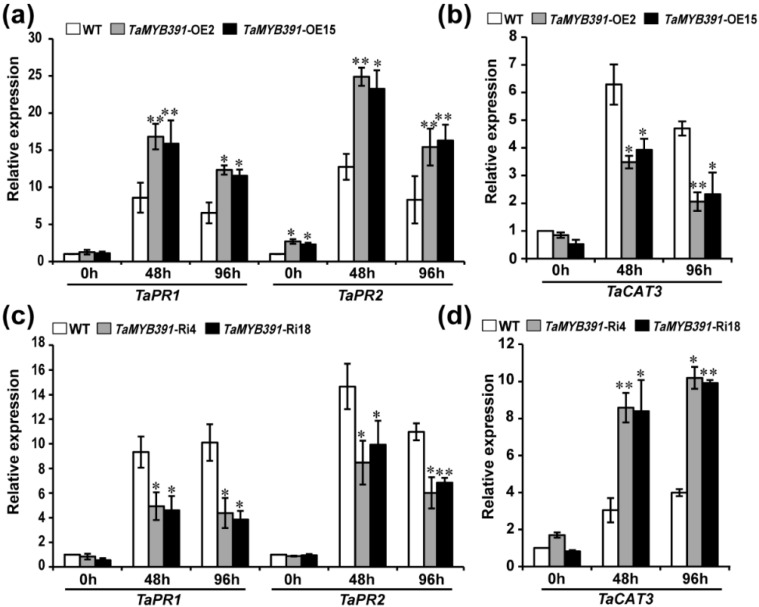
Relative expression of pathogenesis-related (PR) and ROS-scavenging genes in *TaMYB391*-overexpressing and *TaMYB391*-RNAi plants challenged with *Pst*. (**a**) The transcript levels of *TaPR1* and *TaPR2* in *TaMYB391*-overexpressing and WT plants infected with CYR31. (**b**) The transcript levels of *TaCAT3* in *TaMYB391*-overexpressing and WT plants challenged with CYR31. (**c**) The transcript levels of *TaPR1* and *TaPR2* in *TaMYB391*-RNAi and WT plants inoculated with CYR23. (**d**) The transcript levels of *TaCAT3* in *TaMYB391*-RNAi and WT plants infected with CYR23. Relative expression of these genes was computed by the comparative threshold (2^−ΔΔCt^) method. The data were normalized with the transcripts of the reference gene, *TaEF-1α*, and expressed as fold changes relative to the control (WT) at 0 h. Data obtained from control plants at 0 hpi were normalized as 1. Values represent the means ± SEs (*n* = 3). Significant differences between *TaMYB391*-overexpressing or *TaMYB391*-RNAi and control plants determined by Student’s *t*-test are indicated by asterisks. *, *p* < 0.05; **, *p* < 0.01.

Pathogenesis-related (PR) genes play diverse roles in host–pathogen interactions [44,45,46]. *PR1* and *PR2* are the marker genes for SA-mediated activation of SAR [47]. The transcriptional accumulation of *TaPR1*, *TaPR2* and a ROS-scavenging catalase gene *TaCAT3* were quantified in *TaMYB391*-overexpressing and *TaMYB391*-RNAi wheat transgenic lines using RT-qPCR. The RT-qPCR results revealed that the transcript levels of *TaPR1* and *TaPR2* were significantly increased in the *TaMYB391*-overexpressing lines compared to WT controls when inoculated with CYR31 (Figure 5a). Conversely, the expression levels of *TaCAT3* in the *TaMYB391*-overexpressing lines were significantly decreased compared to WT when inoculated with CYR31 (Figure 5b). On the other hand, the transcriptional accumulation of *TaPR1* and *TaPR2* genes in the *TaMYB391*-RNAi lines was significantly decreased compared to control plants when inoculated with CYR23 (Figure 5c), whereas the transcript levels of *TaCAT3* were significantly increased compared to the WT when inoculated with CYR23 (Figure 5d). Together these results demonstrated that *TaMYB391* is involved in the regulation of certain defense-related and ROS-scavenging genes.

### 2.10. Pst Growth and H_2_O_2_ Accumulation Were Significantly Affected in TaMYB391-Overexpressing and TaMYB391-RNAi Transgenic Lines

**Figure 6:**
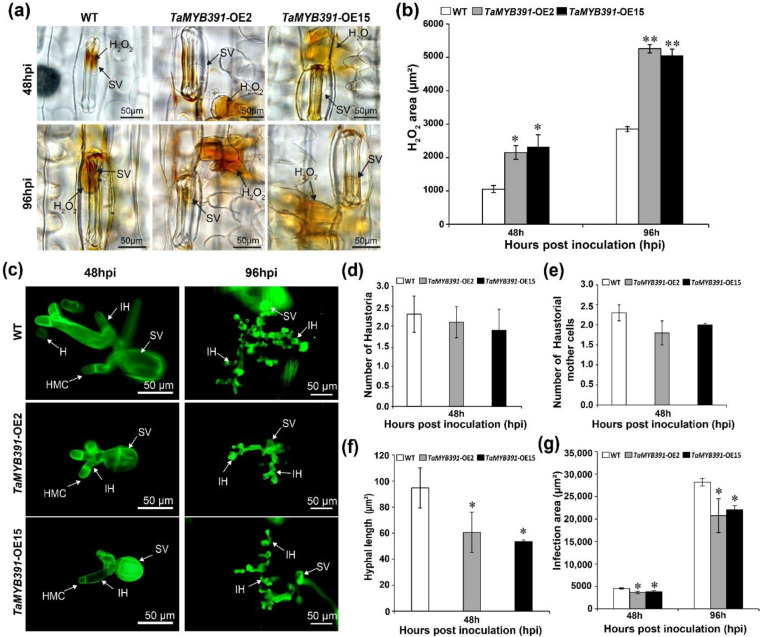
The overexpression of *TaMYB391* improves wheat resistance to the virulent *Pst* race CYR31, while *Pst* growth is diminished. (**a**) H_2_O_2_ accumulation around the infection site was detected under a BX-51 microscope (Olympus, Tokyo, Japan). For H_2_O_2_ burst detection, wheat leaves inoculated with *Pst* CYR31 were sampled at 48 and 96 hpi. These samples were then stained with 3,3-diamino-benzidine (DAB). SV, substomatal vesicle; (**b**) H_2_O_2_ accumulation was quantified using DP-BSW software (Olympus, Tokyo, Japan) by measuring the area where DAB is accumulated at the infection site. (**c**) Fungal structures in *TaMYB391*-overexpressing and control plants challenged with the virulent *Pst* race CYR31. Leaves inoculated with *Pst* were sampled at 48 and 96 hpi. Samples were then stained with wheat germ agglutinin (WGA) and fungal growth was detected using an Olympus BX-51 microscope (Olympus, Tokyo, Japan). SV, substomatal vesicle; HMC, haustorial mother cell; IH, infection hypha; H, haustoria. (**d**) The average number of haustoria per infection site. (**e**) Average number of haustorial mother cells per infection site. (**f**) Hyphal length as measured from the juncture of the hypha and substomatal vesicle to the tip of the hypha. (**g**) The colony area per each infection site. Data were computed from three biological replications and 50 infection sites. Values represent the means ± SEs. The significant difference between *TaMYB391*-overexpressing and control plants is indicated by asterisks, which was estimated using Student’s *t*-test. *, *p* < 0.05; **, *p* < 0.01.

**Figure 7:**
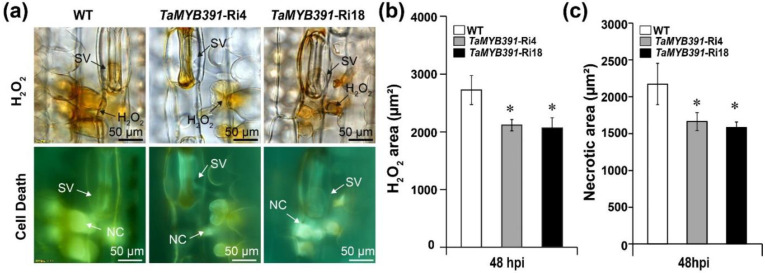
RNAi-mediated silencing of *TaMYB391* decreased wheat resistance to avirulent *Pst* race CYR23 infection. (**a**) H_2_O_2_ accumulation and cell death around the infection site were detected under a BX-51 microscope (Olympus, Tokyo, Japan). For H_2_O_2_ burst and necrosis detection, *Pst* CYR23-inoculated leaves were sampled at 48 hpi. These samples were then stained with 3,3-diamino-benzidine (DAB). SV, substomatal vesicle; NC, necrotic cell. (**b**) H_2_O_2_ accumulation was quantified using DP-BSW software by measuring the area where DAB is accumulated at the infection site. (**c**) The area of cell death was quantified by calculating the fluorescence area. Data were computed from three biological replications and 50 infection sites. Values represent the means ± SEs. The significant differences between *TaMYB391*-RNAi and control plants are indicated by asterisks, which were estimated using Student’s *t*-test. *, *p* <0.05.

**Figure 8:**
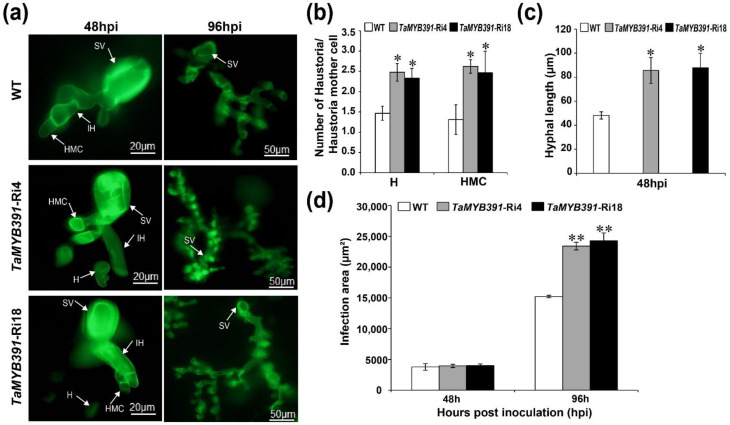
RNAi-mediated silencing of *TaMYB391* enhances *Pst* growth. (**a**) Fungal structures in *TaMYB391*-RNAi and control plants challenged with the avirulent *Pst* race CYR23. Leaves inoculated with *Pst* were sampled at 48 and 96 hpi. Samples were then stained with wheat germ agglutinin (WGA) for fungal growth detection. Microscopy detection of different structures of *Pst* was performed by using an Olympus BX-51 microscope. SV, sub-stomatal vesicle; IH, infection hypha; HMC, haustorial mother cells; H, haustoria, WT, wild type. (**b**) The average number of haustoria and haustorial mother cells per infection site. (**c**) Hyphal length as measured from the juncture of the hypha and substomatal vesicle to the tip of the hypha. A DP-BSW tool (Olympus) was used to compute the hyphal length. (**d**) The colony area per each infection site as measured by the DP-BSW tool (Olympus). Data were computed from three biological replications and 50 infection sites. Values represent the means ± SEs. Significant differences between *TaMYB391*-RNAi lines and control plants were estimated using Student’s *t*-test and indicated by asterisks. *, *p* < 0.05; **, *p* < 0.01.

The phenotypic variation between the WT and *TaMYB391*-overexpressed or under-expressed (*TaMYB391*-RNAi) transgenic lines was further confirmed through histological observation. To clarify the changes in the host resistance level, H_2_O_2_ accumulation and HR-associated cell death around the infection site were observed microscopically and measured by DP-BSW software. The growth of *Pst* was also measured by DP-BSW software. The histological observation results showed that H_2_O_2_ production in the *TaMYB391*-overexpressing transgenic lines showed a substantial increase in comparison to the WT controls (Figure 6a,b). As compared to the WT, hyphal length and infection area were significantly decreased in the *TaMYB391*-overexpressing lines (Figure 6c,f,g). There were no significant differences between WT and *TaMYB391*-overexpressing transgenic lines in the number of haustoria and haustorial mother cells (Figure 6c–e). Our data suggested that overexpression of *TaMYB391* improves the resistance of wheat to *Pst*. On the other hand, as compared to the WT controls, H_2_O_2_ accumulation was significantly decreased in the *TaMYB391*-RNAi lines (Figure 7a,b). Moreover, the necrotic area in the RNAi lines was significantly lower than in the WT controls (Figure 7a,c). Furthermore, the number of haustoria and haustorial mother cells, and hyphal length in the *TaMYB391*-RNAi lines were significantly increased compared to WT, at 48 hpi (Figure 8a–c). In addition, the infection area in the *TaMYB391*-RNAi line was significantly higher than in the WT controls, at 96 hpi (Figure 8a,d). Therefore, our results strongly suggest that RNAi-mediated silencing of *TaMYB391* enhances wheat susceptibility to *Pst*.

## 3. Discussion

Transcription factors in general, and MYB TFs in particular, play pivotal roles in plant disease resistance. Many MYB TFs have been reported for their positive roles in disease resistance in a variety of plant species. Here we reported the functional role of an R2R3 MYB TF, *TaMYB391*, during wheat–*Pst* interaction through gain- and loss-of-function approaches. RT-qPCR analysis demonstrated that the expression of *TaMYB391* is associated with plant immunity against the biotrophic fungal pathogen *Pst*. Similarly, some MYB genes which are reported to participate in defense responses were induced after infection with different pathogens [23,25,26,27]. Furthermore, *TaMYB391* was also significantly induced by exogenous treatment of SA and ABA. Our sub-cellular localization assay indicated that TaMYB391 is a nuclear-targeted protein. In previous studies, many MYB TFs (e.g., *AtMYB30*, *TaPIMP1*, *TaMYB4*, *TaPIMP2* and *TaRIM1*) have been reported to be localized in the nucleus [23,24,25,26,48].

In plants, the hypersensitive response (HR), a kind of PCD, is usually associated with disease resistance. To dissect the role of *TaMYB391* in HR-related PCD, we transiently overexpressed *TaMYB391* in *N. benthamiana*. The results demonstrated that *TaMYB391* is involved in inducing PCD directly. This is supported by increased electrolyte leakage. Accumulated evidence has indicated that a number of plant genes regulate the induction of HR-related PCD in plants. For instance, *AtMYB30* is a well-known regulator of hypersensitive cell death [49]. Overexpression of *Pti1* in tobacco accelerates HR in response to *P. syringae* pv. *tabaci* [50]. Antisense expression of *hsr203* has also been shown to accelerate HR in tobacco in response to different pathogens [41]. ROS accumulation was observed in *TaMYB391* agroinfiltrated samples. Meanwhile, the transcriptional accumulation of HR-specific marker genes and defense-related marker genes were significantly up-regulated. Our results indicated that HR-associated PCD triggered by *TaMYB391* is correlated with the innate immune response.

In wheat, exogenous application of SA and ABA also increased the expression of *TaMYB391*. *PR1* and *PR2* are considered to be marker genes for SA-mediated activation of SAR [47]. The transcript levels of *TaPR1* and *TaPR2* in the *TaMYB391*-overexpressing plants were significantly up-regulated, whereas in the *TaMYB391*-RNAi lines the expression was repressed compared to WT. Plant hormones have been reported to play a significant role in plant biotic stress response [29,51]. SA is usually involved in biotrophic and hemibiotrophic pathogen defense signals [30,32]. ABA is reported to take part in both biotic and abiotic stress signals [52]. SA-mediated disease resistance against biotrophic pathogens was generally negatively regulated by ABA [53]. Some MYB TFs, for example, *BOS1* [54], *AtMYB96* [17,55], *TaPIMP1* [23] and *TaPIMP2* [24] have been proven to participate in hormone signaling networks. Taking these results together, *TaMYB391* appears to activate the innate immune response via SA-mediated defense pathways.

The functional role of *TaMYB391* in the wheat–*Pst* interaction was further validated through the gain- and loss-of-function approach. Expression analysis, phenotypic and microscopic assays indicated that *TaMYB391*-overexpression confers improved resistance to *Pst* infection, while the *TaMYB391*-RNAi lines showed decreased resistance to *Pst* compared to WT wheat plants. The degree of resistance in *TaMYB391*-overexpressing transgenic lines was correlated with reduced fungal growth (hyphal length and infection area), increased H_2_O_2_ accumulation and enhanced expression of defense-related genes. H_2_O_2_ accumulation, necrotic area and expression of defense-related genes in the RNAi lines, on the other hand, showed a significant reduction, while fungal growth was enhanced compared to WT wheat plants. These results suggested that *TaMYB391* is positively involved in wheat resistance response to *Pst* infection. Consistent with our findings, the mutation or silencing of some MYB genes, such as *TaMYB29*, *OsMYB30*, *TaPIMP1*, *TaMYB4*, *TaLHY*, *TaRIM1*, *TaPIMP2*, *MYB115* and *AtMYB15* has been found to enhance susceptibility to different pathogens [18,19,23,24,25,26,27,28,56].

As *PR* genes are reported to play a significant role in defense responses against various pathogens in different plant species [44,45,46], we analyzed the transcriptional accumulation of two *PR* genes (*TaPR1* and *TaPR2*) in *TaMYB391*-overexpressing and *TaMYB391*-RNAi lines when inoculated with *Pst*. The expression levels of these genes in the *TaMYB391*-overexpressing plants were significantly up-regulated, whereas in the *TaMYB391*-RNAi lines the expression was repressed compared to WT. These results demonstrated that *TaMYB391* positively regulates the expression of certain *PR* genes during *Pst* infection. Furthermore, the expression of a ROS-scavenging gene *TaCAT3* was significantly decreased in the *TaMYB391*-overexpressing plants, while being significantly increased in the *TaMYB391*-RNAi lines compared to WT plants. Previous studies have indicated that MYB TFs regulate the expression of diverse defense-related genes by binding to the respective cis-acting elements in their promoter regions [13,14,16,56,57]. In the future, we plan to identify the downstream target genes of TaMYB391 and clarify how TaMYB391 regulates the expression of *TaPR1*, *TaPR2* and *TaCAT3* during the interaction between wheat and *Pst*.

In conclusion, we identified an R2R3 MYB TF from wheat, *TaMYB391*, which acts as a positive regulator of hypersensitive response cell death. The expression of *TaMYB391* was induced upon infection with *Pst* and exogenous application of SA and ABA. SA-dependent defense-related genes were also induced by *TaMYB391* in *N. benthamiana*. Results obtained from overexpression and RNAi assays demonstrated that *TaMYB391* positively regulates the resistance of wheat to *Pst*. Together, our data demonstrate that *TaMYB391* acts as a positive regulator of HR and contributes to wheat resistance to *Pst* by modulating the defense-related genes, probably through SA-signaling pathways.

## 4. Materials and Methods

### 4.1. Plant Materials, Fungal Isolates and Inoculation/Treatments

Two wheat (*T. aestivum* L.) cultivars, Suwon11(Su11) and Fielder were used. Two *Pst* isolates, CYR23 and CYR31, were also used in our study. Su11, carrying a *YrSu* resistance gene, is susceptible to CYR31 and resistant to CYR23 [40]. Su11 was used to determine the relative transcription of *TaMYB391* after *Pst* infection and exogenous hormones treatment. Su11 was also used to amplify the cDNA sequences of *TaMYB391* and to perform the sub-cellular localization assays. Fielder is often used to introduce genes of interest for RNAi and overexpression due to its high transformation success rate [58]. Fielder, carrying *Yr6* and *Yr20* resistance genes, is susceptible to CYR31 and resistant to CYR23 [5,59]. In this study, Fielder was used as receptor material to generate *TaMYB391*-overexpressed and *TaMYB391*-RNAi transgenic plants. Wheat leaves challenged with the *Pst* isolates or water (distilled and sterile) were sampled at different time points for RNA isolation and histological study. Tobacco (*Nicotiana benthamiana*) was used for transient overexpression of *TaMYB391* and sub-cellular localization assays.

Seedlings of Su11 were also subjected to different hormone treatments. Four hormones were used in this study. Wheat seedlings were sprayed with 100 µM methyl jasmonate (MeJA), 100 µM ethylene (Eth), 100 µM abscisic acid (ABA) and 2 nM salicylic acid (SA) that were all dissolved in 0.1% (*v*/*v*) ethanol. Mock-treated seedlings were sprayed with 0.1% (*v*/*v*) ethanol. Leaf samples were collected at 0, 0.5, 1, 2, 6, 12, 24 and 48 h post-treatment (hpt) for total RNA extraction. Three independent biological replications were carried out for each treatment.

### 4.2. RNA Extraction, cDNA Synthesis and Gene Expression Analysis

A Quick RNA isolation Kit (Huayueyang Biotechnology, Beijing, China) was used for total RNA extraction and was performed according to the manufacturer’s instructions. A spectrometer (NanoDrop) was then used to quantify the isolated RNA. The enzyme DNase I was used to remove the DNA contamination. A RevertAid First Strand cDNA Synthesis Kit (Thermo Scientific, Waltham, MA, USA) was used to reverse the transcription of RNA and synthesize first-strand cDNA from 3 μg of isolated RNA. The transcriptional accumulation of *TaMYB391* was then analyzed using RT-qPCR with specific primers (Table S1). Gene expression was quantified using a ChamQ SYBR qPCR Master Mix (Vazyme, Shanghai, China) with a CFX Connect Real-Time System (Bio-Rad, Hercules, CA, USA). In wheat and *N. benthamiana*, the wheat elongation factor *TaEF-1α* (GenBank accession number: M90077) and *NbActin* (GenBank accession number AY179605) were used as an internal reference to quantify the transcript levels [10,60,61,62,63,64]. The relative transcript levels of specific genes were computed using the comparative 2^−ΔΔCT^ method. Each reaction was replicated three times.

### 4.3. Identification, Cloning and Sequence Analysis of TaMYB391

A 1077 bp nucleotide sequence (accession number TraesCS7D02G514800) was obtained from the cDNA library in our lab. The coding sequence (CDS) of *TaMYB391* was amplified from the cDNA template obtained from Su11 (Table S1). The cDNA sequence of *TaMYB391* was analyzed using NCBI BLAST (http://www.ncbi.nlm.nih.gov/blast/ (accessed on 7 October 2020)).) and the ORF finder software at NCBI. The conserved domain was predicted with Pfam (http://pfam.sanger.ac.uk/ (accessed on 10 October 2020)), PROSITEScan (http://prosite.expasy.org/scanprosite/ (accessed on 10 October 2020)) and InterproScan (http://www.ebi.ac.uk/Tools/pfa/iprscan/ (accessed on 10 October 2020)). The nuclear localization signal (NLS) region was predicted with a cNLS Mapper (http://nls-mapper.iab.keio.ac.jp/cgi-bin/NLS_Mapper_form.cgi (accessed on 13 October 2020)). DNAMAN8.0 (Lynnon BioSoft, San Ramon, CA, USA) was used to perform multiple sequence alignments. A phylogenic tree of the amino acid sequences was generated using MEGA7.0 software.

### 4.4. Subcellular Localization of TaMYB391 in Wheat and N. benthamiana

The subcellular localization assay was performed to detect where *TaMYB391* is located in the plant cell. Wheat protoplasts and *N. benthamiana* were used for this assay. The full-length CDS of *TaMYB391* was subcloned into p16318hGFP or pCAMBIA:1302 vectors. Wheat protoplasts were extracted from the healthy fresh leaves of two-week-old wheat seedlings as described [65]. The p16318hGFP–*TaMYB391* fusion or GFP construct were independently introduced into wheat protoplasts, as previously described [65]. The transformed wheat protoplasts were incubated in a dark chamber at 24 °C for 24-36 h and then observed with an Olympus FV1000 confocal laser microscope with a 488nm filter (Olympus, Tokyo, Japan). The localization of *TaMYB391* was further confirmed by introducing pCAMBIA1302:*TaMYB391* fusion into *Agrobacterium tumefaciens* strain GV3101 through electroporation, which was then agroinfiltrated into 4-week-old *N. benthamiana* leaves as described [66]. Infiltrated *N. benthamiana* leaves were kept in a growth chamber with a 16h/8h photoperiod at 25 °C for 2 to 3 days. Fluorescence signals were then observed and photographed with an Olympus FV1000 confocal laser microscope with a 488 nm filter (Olympus, Tokyo, Japan).

### 4.5. Transient Overexpression of TaMYB391 in N. benthamiana

A recombinant plasmid, PVX: *TaMYB391*, was constructed by cloning full length CDS without the stop codon into the pGR106 vector. The reconstructed vectors, PVX: *TaMYB391*, PVX: *Pst322* (positive control) and PVX: GFP or empty vector (EV) (negative control), were transformed into *A. tumefaciens* strain GV3101 using electroporation, and then agroinfiltrated into 4-weeks-old *N. benthamiana* leaves as described [66]. The agroinfiltrated leaves were sampled at 0, 48, 72, 96 and 120 h post-agroinfiltration (hpa) for RNA isolation, and then analyzed by RT-qPCR. To detect ROS accumulation, the agroinfiltrated leaves were sampled at 24 hpa, and then stained with 3,3-diamino-benzidine (DAB). Symptom development was monitored at 3–8 dpa. Three biological replications were performed, and each assay consisted of three plants with three leaves.

### 4.6. Measurement of Electrolyte Leakage

The cell death was further confirmed via ion leakage assay, as previously described [67]. Six leaf disks, each 1 cm in diameter, were punched from PVX: *TaMYB391* and GFP agroinfiltrated leaves and immersed in 5 mL distilled water for 5 h. After 5 h in distilled water, the ‘value A’ was obtained by measuring the conductivity of the solution with a conductivity meter (FE32 Five Easy; Mettler-Toledo, Shanghai, China). Then, the solution with the leaf disks was sealed and subjected to boiling for 20 min. After letting the solution cool, its conductivity was again measured to obtain ‘value B’. Ion leakage was then computed as percent leakage; (value A/value B) × 100%. This assay was repeated three times.

### 4.7. Generation of Transgenic Lines

To produce *TaMYB391*-overexpressing transgenic wheat plants, the CDS of *TaMYB391* was sub-cloned into the pCUB vector [68], and the pCUB: *TaMYB391* construct was generated. The expression of *TaMYB391* was driven by the maize ubiquitin promoter. pCUB: *TaMYB391* was then transformed into wheat variety Fielder by an *Agrobacterium*-mediated wheat transformation system. Positive *TaMYB391*-overexpressed transgenic plants were validated through PCR using specific primers (Figure S4a; Table S1). The wild-type (WT) wheat variety Fielder was used as a negative control during the PCR check. The second leaves of T_2_ transgenic lines were challenged with the virulent *Pst* race, CYR31. Samples from *Pst*-infected leaves were collected for RNA isolation and histological observation.

To generate the *TaMYB391*-RNAi construct, the 200 bp specific fragment of *TaMYB391* was amplified and inserted into the pC336 vector [69] using gateway cloning technology. The *TaMYB391*-RNAi construct contains an inverted repeat. A double-stranded (ds) RNA sequence with a hairpin structure was produced after transcription from the construct to induce the silencing of *TaMYB391*. *TaMYB391*-RNAi vector was then transformed into wheat variety Fielder by an *Agrobacterium*-mediated wheat transformation system. Positive transformants in the T_1_ generation were detected by PCR using universal primers (Table S1). The second leaf of the T_2_ generation was inoculated with the avirulent *Pst* race CYR23 and infected leaves were sampled for RNA isolation and histological observation.

### 4.8. H_2_O_2_ Accumulation and Histological Observation of Fungal Growth

Wheat leaves inoculated with *Pst* isolates, CYR23 or CYR31, were sampled at 48 and 96 hpi and stained to detect the accumulation of H_2_O_2_ and fungal structures. H_2_O_2_ accumulation was examined by staining samples with DAB. Samples were then stained with wheat germ agglutinin stain (WGA) (Invitrogen, Waltham, MA, USA) for histochemical analysis [70]. During the wheat–*Pst* interaction, the formation of a substomatal vesicle is considered to be an effective penetration site of *Pst*. At least 30–50 infection sites were examined for each treatment to assess the H_2_O_2_ accumulation, necrotic area and various fungal structures. Necrotic cells around the infection site, H_2_O_2_ accumulation and fungal structures, such as hypha, haustoria mother cells and haustoria, and infection area were observed with a BX-51 microscope (Olympus, Tokyo, Japan), and their corresponding lengths and areas were estimated using DP-BSW software. The experiment was repeated three times.

### 4.9. Statistical Analysis

All data were subjected to analysis with Microsoft Excel. Microsoft Excel was used to calculate mean values and standard errors. Student’s *t*-test was used to determine the significant differences between control and treatment groups or between time courses. Significant difference was measured by probability (*p*) values (*p* < 0.05 or *p* < 0.01).

## Data Availability

Not applicable.

## Supplementary Materials

The following supporting information can be downloaded at: https://www.mdpi.com/article/10.3390/ijms232214070/s1.
