# Supplementary material for: A R2R3 MYB Transcription Factor, TaMYB391, Is Positively Involved in Wheat Resistance to Puccinia striiformis f. sp. tritici
Source: Int J Mol Sci. 2022 Nov 15;23(22):14070. doi: 10.3390/ijms232214070 (PMC9693031; doi:10.3390/ijms232214070)
Supplement: Supplementary file 1 [file ijms-23-14070-s001.zip › ijms-1912897-supplementary.pdf]

Supplementary Data

A R2R3 MYB Transcription Factor, *TaMYB391*, Is Positively Involved in Wheat Resistance to *Puccinia striiformis* f. sp. *tritici*

Mehari Desta Hawku [1,2,†], Fuxin He [1,†], Xingxuan Bai [1], Md Ashraful Islam [1], Xueling Huang [1], Zhensheng Kang [1,*] and Jun Guo [1,*]

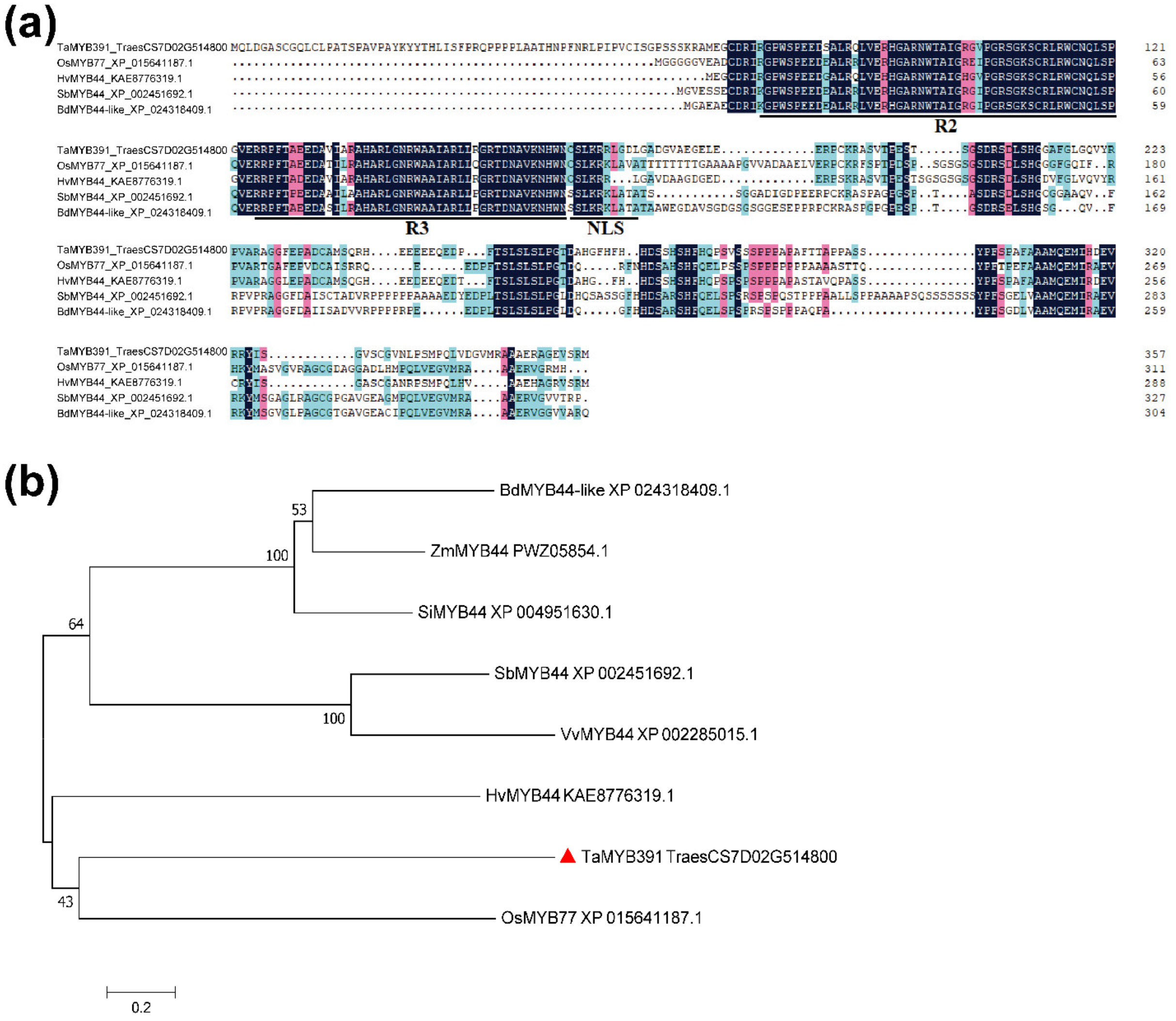

Figure S1. Multisequence alignment and phylogenetic analysis of the deduced amino acid sequences of TaMYB391 with other plant species. (**a**) Multisequence alignment of *TaMYB391* with other plant species. Comparison of amino acid sequences of *Triticum aestivum* (*TaMYB391*) with MYB genes from *Oryza sativa* (GenBank accession No. XP_015641187.1), *Hordeum vulgare* (GenBank accession No. KAE8776319.1), *Sorghum bicolor* (GenBank accession No. XP_002451692.1) and *Brachypodium distachyon* (GenBank accession No. XP_024318409.1). Conserved residues through all organisms are shown in black (100%), pink (75–100%) and light blue (50–75%), respectively. Sequences alignment was performed using DNAMAN8.0 (Lynnon Biosoft, San Ramon, QC, Canada). (**b**) Phylogenetic analysis of the deduced amino acid sequences of TaMYB391 with other plant species. Ta, *Triticum aestivum*; Os, *Oryza sativa;* Bd, *Brachypodium distachyon;* Hv, *Hordeum vulgare;* Sb, *Sorghum bicolor;* Zm, *Zea mays*; Si, *Setaria italica*; Vv, *Vitis vinifera*.

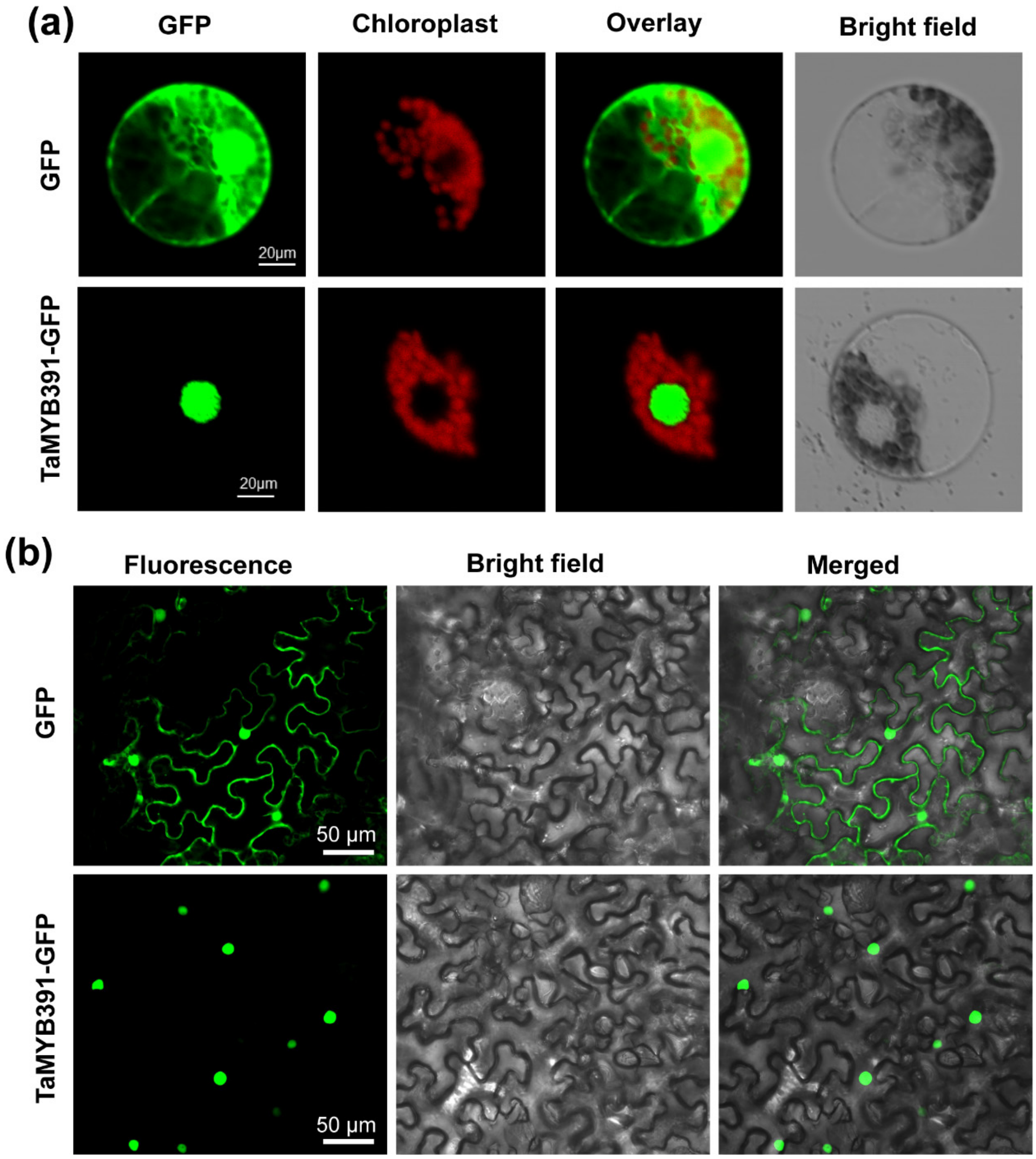

Figure S2. *TaMYB391* is localized in the nucleus. (**a**) p16318hGFP:TaMYB391 fused proteins were overexpressed in wheat protoplasts through PEG-mediated transfection method. GFP (green fluorescence protein) was used as a control. (**b**) pCAMBIA1302:TaMYB391-GFP fused proteins were overexpressed in tobacco through *Agrobacterium*-mediated transformation. The constructs, GFP or TaMYB391-GFP, were first introduced into *A. tumefaciens* then agroinfiltrated into *N. benthamiana* leaves. GFP signals were observed with an Olympus FV1000 confocal microscope with 488 nm filter.

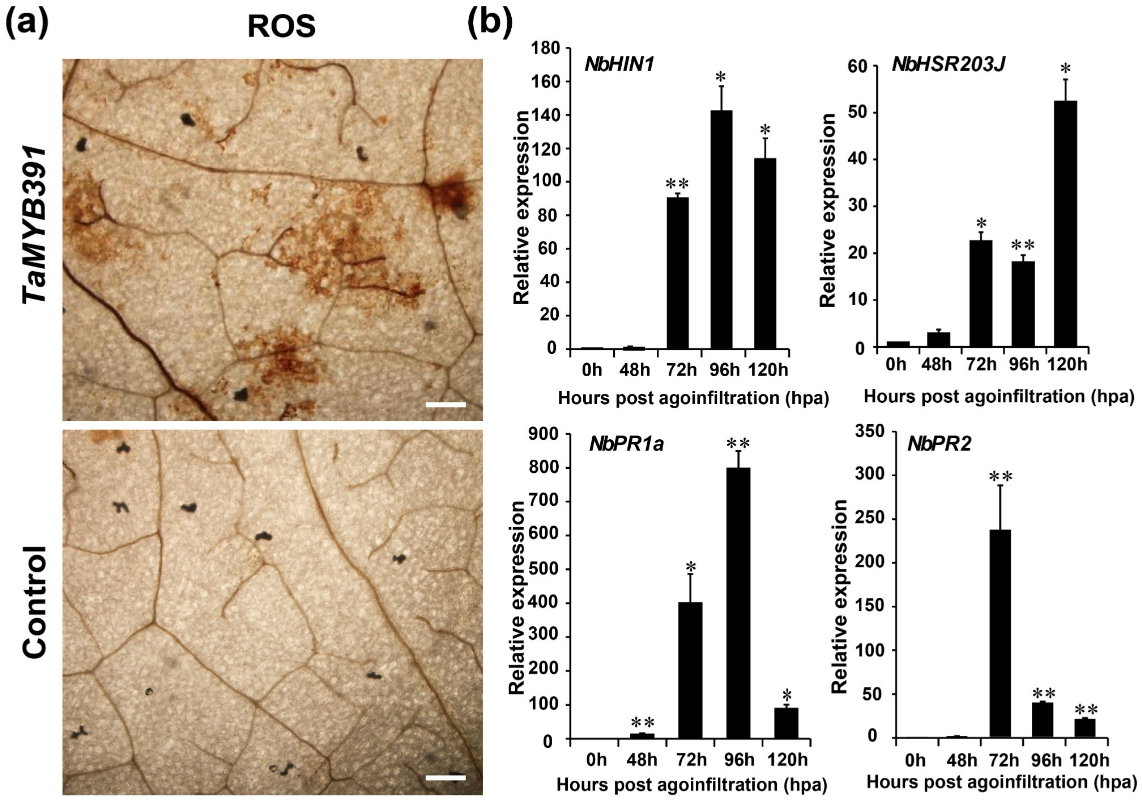

Figure S3. Transient overexpression of *TaMYB391* induces ROS accumulation and expression of HR- and defense-related marker genes in *N. benthamiana*. *TaMYB391* triggers plant immunity response in *N. benthamiana*. (**a**) Reactive oxygen species (ROS) accumulation in *N. benthamiana*. *A. tumefaciens* cells carrying PVX: *TaMYB391* or PVX: *GFP* were infiltrated into *N. benthamiana* leaves. *A. tumefaciens* cells carrying PVX: *GFP* were used as control. Bar, 200μm. (**b**) Relative expression of hypersensitive-response (HR) specific- and defense-related- marker genes in *N. benthamiana*. *NbActin* was used as the internal reference gene. Relative expression of genes tested was normalized to *NbActin*. The quantitative RT-PCR values are presented as fold changes relative to that in control leaves at the same time. Means and SEs were computed from three replicates. The statistical analyses were performed with Student's *t*-test. Bars indicate ± SE. *, $p < 0.05$; **, $p < 0.01$.

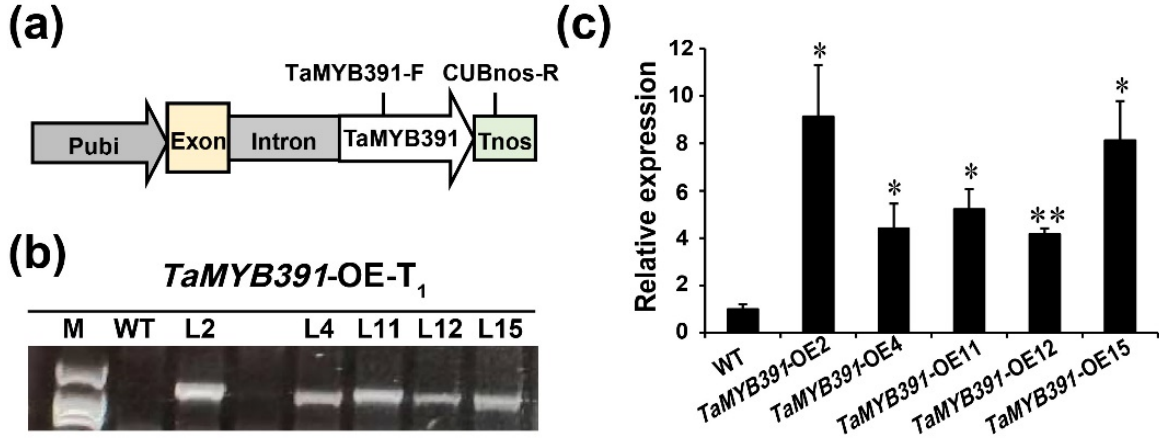

Figure S4. PCR and RT-qPCR assays to identify the positive T_1 *TaMYB391*-overexpression transgenic lines. (**a**) Scheme of the transformation vector pCUB: *TaMYB391*. The expression of *TaMYB391* was driven by Pubi and terminated by Tnos. *Pubi*: maize ubiquitin promoter; *Tnos*: terminator of *A. tumefaciens* nopaline synthase gene. The primers used to check the positive transformants by PCR are indicated on the top of the schema. (**b**) Five successfully transformed transgenic lines were detected in T_1 generation using PCR. M: molecular marker; WT: wild type. (**c**) Transcription levels of five positive lines in T_1 generation analyzed through RT-qPCR. The data were normalized with the transcripts of the reference gene, *TaEF-1α*, and expressed as fold changes relative to WT. Means and SEs were computed from three replicates. The statistical analyses were performed with Student's *t*-test. Bars indicate ± SE. *, $p < 0.05$; **, $p < 0.01$.

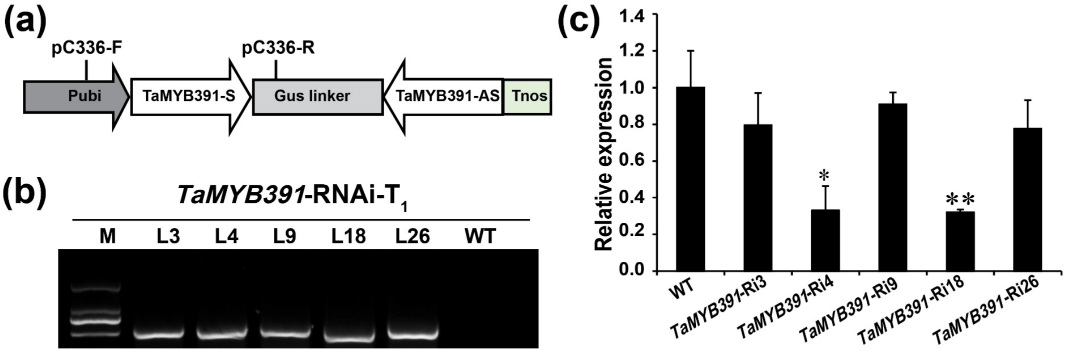

Figure S5. PCR and RT-qPCR assays to identify the positive T_1 *TaMYB391*-RNAi transgenic lines. (**a**) Scheme of the *TaMYB391*-RNAi vector. The specific fragment derived from *TaMYB391* in the sense (S) and antisense (AS) orientations was constructed such that the gus linker sequence was inserted between the S and AS sequences. The expression was driven by Pubi and terminated by Tnos. *Pubi*: maize ubiquitin promoter; *Tnos*: terminator of *A. tumefaciens* nopaline synthase gene. The primers used to check the positive transformants by PCR are indicated on the top of the schema (**b**) Five successfully transformed transgenic lines were detected in T_1 generation using PCR. M, molecular marker; WT, wild type. (**c**) Transcription levels of five positive lines in T_1 generation analyzed through RT-qPCR. The data were normalized with the transcripts of the reference gene, *TaEF-1α*, and expressed as fold changes relative to WT. Means and SEs were computed from three replicates. The statistical analyses were performed with Student's *t*-test. Bars indicate ± SE. *, $p < 0.05$; **, $p < 0.01$.

Table S1. Primers used in this study.

Purpose	Name	Sequence 5' to 3'
Gene amplification	TaMYB391-F	ATGCAGTTGGATGGGGCCTCGT
	TaMYB391-R	TCACCGCATCCTCGAGACTTCGC
Cloning of TaMYB391 to CUB vector for overexpression in wheat	CUB-TaMYB391-F	caggtcgactctagaggatccATGCAGTTGGATGGGGCC
	CUB-TaMYB391-R	gagctcggtacccggggatccTCACTTATCATCATCATCCTTATA ATCTCCCTTATCATCATCATCCTTATAATCTCCCTTATC ATCATCATCCTTATAATCCCGCATCCTCGAGACTTCG
For validating positive transformant of TaMYB391-Overexpression	TaMYB391-F	ATGCAGTTGGATGGGGCC
	CUBnos-R	TTTGAACGATCGGGGAAATTC
Cloning of TaMYB391-RNAi fragment to p336 vector	pC336-TaMYB391-F	TTCGAGCCGGCGGACTGC
	pC336-TaMYB391-R	GGCGCGGTGGTGAACGCT
For validating the positive transformants of TaMYB391-RNAi fragment	pc336-F	TTTAGCCCTGCCTTCATACG
	pc336-R	CACGCAAGTCCGCATCTTCA
RT-qPCR in wheat	TaMYB391-RT-qPCR-F	CATGAGCCAGCGACACGA
	TaMYB391-RT-qPCR-R	CGGCGACGAGGAAACAGA
	TaPR1-F	GAGAATGCAGACGCCCAAGC
	TaPR1-R	CTGGAGCTTGCAGTCGTTGATC
	TaPR2-F	AGGATGTTGCTTCCATGTTTGCCG
	TaPR2-R	AAGTAGATGCGCATGCCGTTGATG
	TaCAT-F	GCCCAAGTGCTCCCACCACAACA
	TaCAT-R	TGAGGGTGCGGGAGGGGATG
	TaEF-1α-F	TGGTGTCATCAAGCCTGGTATGGT
	TaEF-1α-R	ACTCATGGTGCATCTCAACGGACT
Biomass ratio analysis	TaEF-1α-F	TGGTGTCATCAAGCCTGGTATGGT
	TaEF-1α-R	ACTCATGGTGCATCTCAACGGACT
	PstEF-F	TTCGCCGTCCGTGATATGAGACAA
	PstEF-R	ATGCGTATCATGGTGGTGGAGTGA
RT-qPCR in *N. benthamiana*	NbHIN1-F	CCAACTTGAACGGAGCCTATTA
	NbHIN1-R	AGGCATCCAAAGAGACAACTAC
	NbHSR203J-F	ACGCAGATTTCAACCGAGTAT
	NbHSR203J-R	GCCAGTCGCATTGGAGATAA
	NbPR1a-F	CCGCCTTCCCTCAACTCAAC
	NbPR1a-R	GCACAACCAAGACGTACTGAG
	NbPR2-F	AGGTGTTTGCTATGGAATGC
	NbPR2-R	TCTGTACCCACCATCTTGC
Overexpression in Tobacco	TaMYB391-PVX-F	tcagcaccagctagcatcgatATGCAGTTGGATGGGGCC
	TaMYB391-PVX-R	aaccgttcatcggcggtcgacCCGCATCCTCGAGACTTCG
Subcellular localization in wheat	TaMYB391-p16318hGFP-F	gacgatatctctagaggatccATGCAGTTGGATGGGGCC
	TaMYB391-p16318hGFP-R	gcccttgctcaccatggatccCCGCATCCTCGAGACTTCG
Subcellular localization in tobacco	TaMYB391-1302-F	CATGGTAGATCTGACTAGTATGCAGTTGGATGGGGCC
	TaMYB391-1302-R	GCCCTTGCTCACCATCCTAGGCCGCATCCTCGAGACTT CG
